# Supplementary material for: SMARCA4 deficient tumours are vulnerable to KDM6A/UTX and KDM6B/JMJD3 blockade
Source: Nat Commun. 2021 Jul 14;12:4319. doi: 10.1038/s41467-021-24618-3 (PMC8280185; doi:10.1038/s41467-021-24618-3)
Supplement: Supplementary file 1 — Supplementary Information [file 41467_2021_24618_MOESM1_ESM.pdf]

SMARCA4 deficient tumours are vulnerable to KDM6A/UTX and KDM6B/JMJD3 blockade

Octavio A. Romero[1#*], Andrea Vilarrubi[1#], Juan J. Alburquerque-Bejar[1], Antonio Gomez[2], Alvaro Andrades[3,4], Deborah Trastulli[5], Eva Pros[1], Fernando Setien[1], Sara Verdura[5], Lourdes Farré[6], Juan F. Martín-Tejera[6], Paula Llabata[1], Ana Oaknin[7], Maria Saigi[1,8] Josep M. Piulats[8], Xavier Matias-Guiu[9], Pedro P. Medina[3,4], August Vidal[9,10], Alberto Villanueva[6,10], Montse Sanchez-Cespedes[1*]

Author affiliations:

[1]Cancer Genetics Group, Josep Carreras Leukaemia Research Institute (IJC), Badalona, Barcelona, Spain. [2]Rheumatology Research Group, Vall d'Hebron Research Institute, Barcelona, Spain. [3]Centre for Genomics and Oncological Research (GENYO), Granada, Spain. [4]Department of Biochemistry and Molecular Biology III, University of Granada, Granada, Spain.[5]Genes and Cancer Group, Cancer Epigenetics and Biology Program (PEBC), Bellvitge Biomedical Research Institute-IDIBELL Barcelona, Spain. [6]Chemoresistance and Predictive Factors Group, Program Against Cancer Therapeutic Resistance (ProCURE), Catalan Institute of Oncology (ICO), Oncobell Program, Bellvitge Biomedical Research Institute (IDIBELL), L'Hospitalet del Llobregat, Barcelona, Spain. [7]Department of Medical Oncology, Vall d'Hebrón Hospital, Barcelona, Spain. [8]Department of Medical Oncology, Catalan Institute of Oncology (ICO), Barcelona, Spain. [9]Department of Pathology, University Hospital of Bellvitge, IDIBELL, CIBERONC, L'Hospitalet del Llobregat, Barcelona, Spain. [10]Xenopat S.L., Parc Cientific de Barcelona (PCB), Barcelona, Spain.

Corresponding authors mscespedes@carrerasresearch.org; oromero@carrerasresearch.org
[#]**These authors contributed equally:** Octavio A. Romero, Andrea Vilarrubi

Supplementary Fig.1

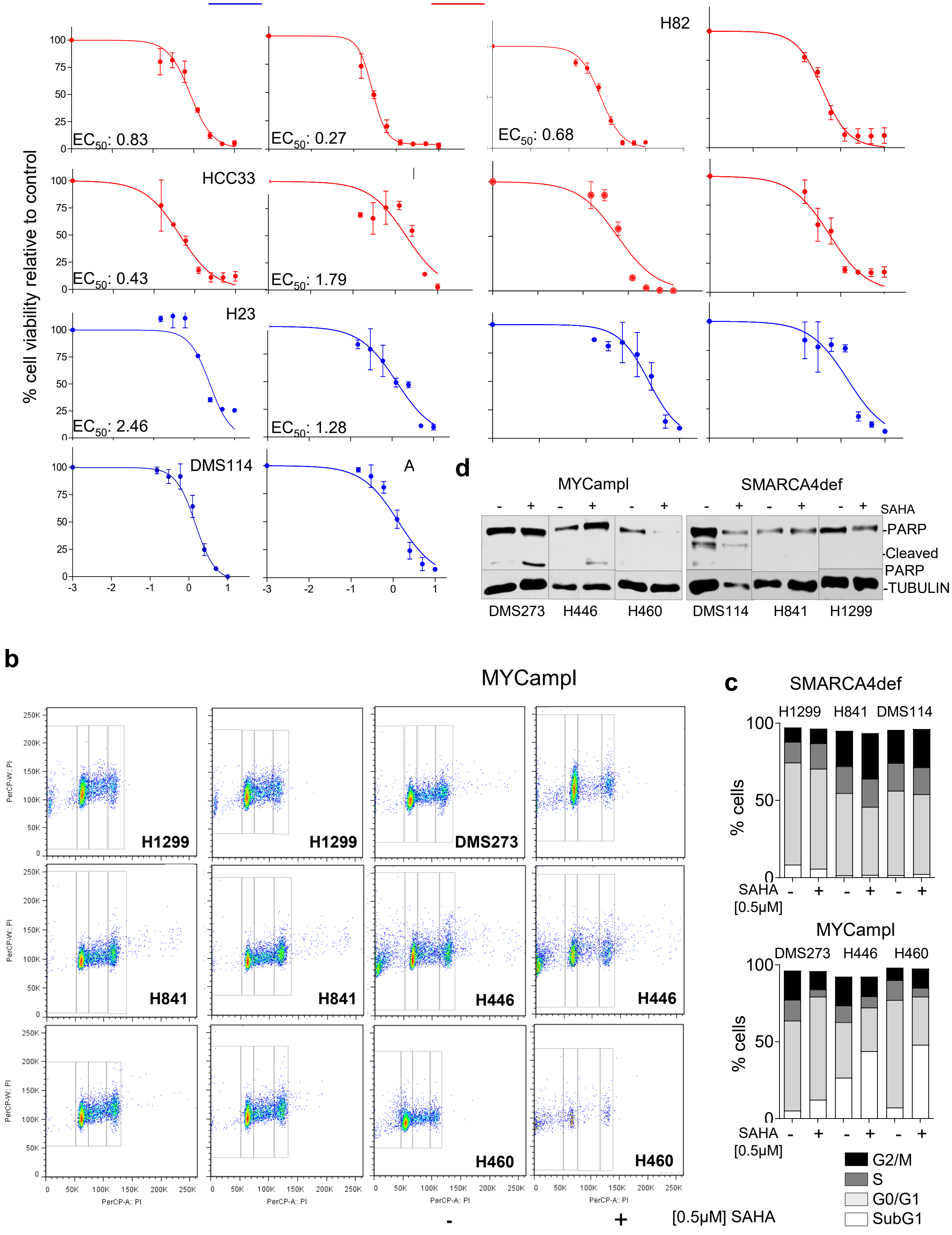

Supplementary Fig.1. SAHA reduces growth in MYCamp, but not in SMARCA4def cancer cells. a, Representative examples of effects on cell viability, measured using MTT assays, in the indicated cell lines after treatment with increasing concentrations of SAHA for 5 days**.** EC_{50}, half maximal effective concentration**.** Error bars, means ± SD of three replicates**. b,** SAHA induced G1 phase arrest in the cell cycle, in MYCamp but not in SMARCA4def cells. Cell cycle distribution was obtained by propidium iodide staining and flow cytometry analysis in cells following treatment with 0.5 µM of SAHA for 48hrs. **c**, Bar graphs depicting the percentage of the indicated cell in each of the cell cycle phases with and without SAHA treatment. **d,** Western blotting depicting the cleavage of PARP in the indicated cells, following the treatment with SAHA or vehicle. A Source Data file is available for this figure.

Supplementary Fig.2

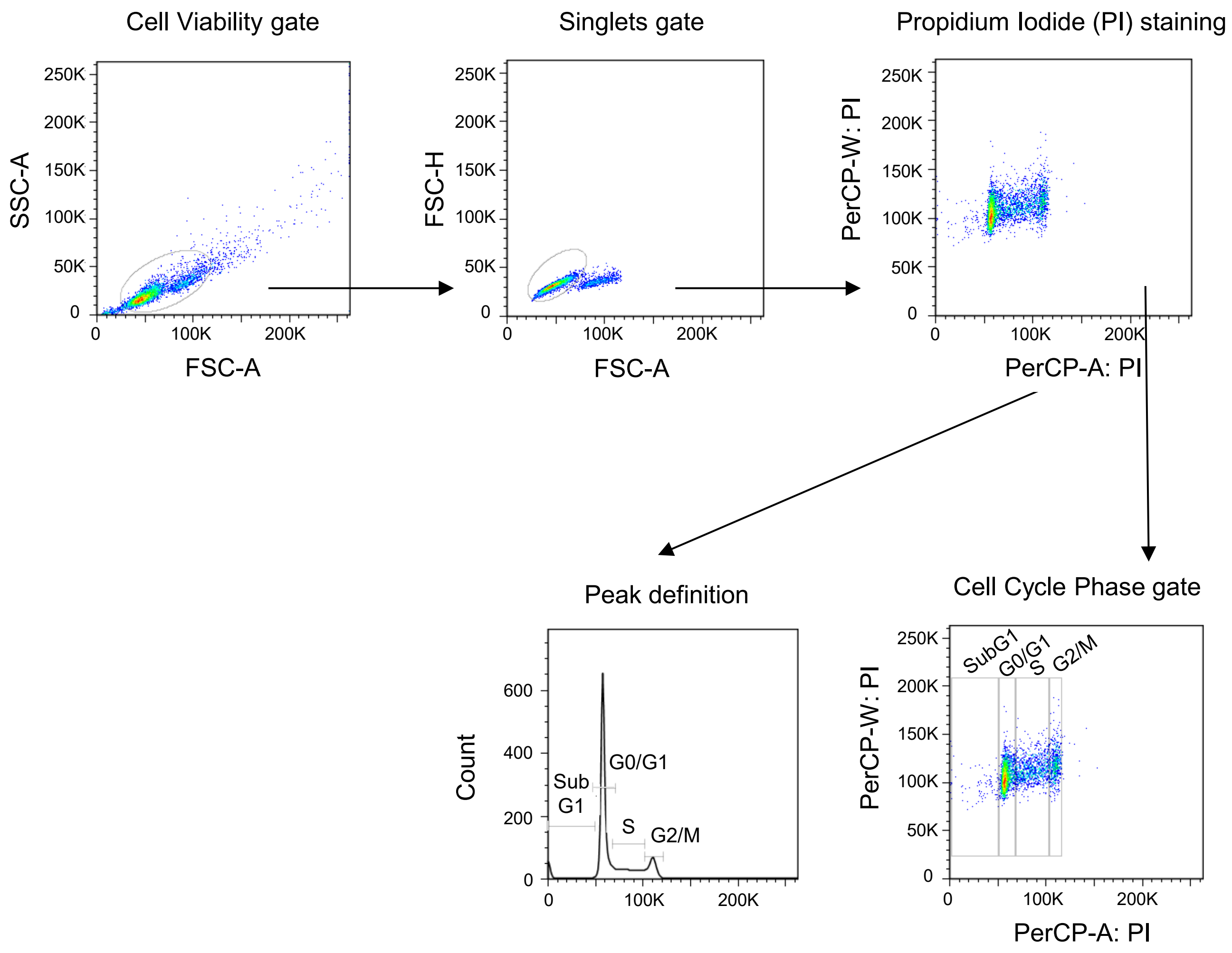

Supplementary Fig.2. Gating strategy for cell cycle analysis of stained MYCamp and SMARCA4def cells with Propidium iodide (PI) as per Supplementary Figure 1b. The gaiting strategy is based on first morphology of viable cells (excludes cell death) > singlets cells (excludes doublets) > PI staining (DNA content estimation). First FACS plot represent relative size (FSC-A) and complexity (SSC-A) of lung cancer cells. Second plot represent FSC-A and FSC-H parameters indicating singlets cells to exclude doublets. Third panel show PI-A and PI-W parameters to detect DNA content of stained cells with Propidium Iodide (PI). Next FACS plot and histogram showing the defined gates to identify SubG1, G0-G1, S and G2-M phase and the associated percentage.

Supplementary Fig.3

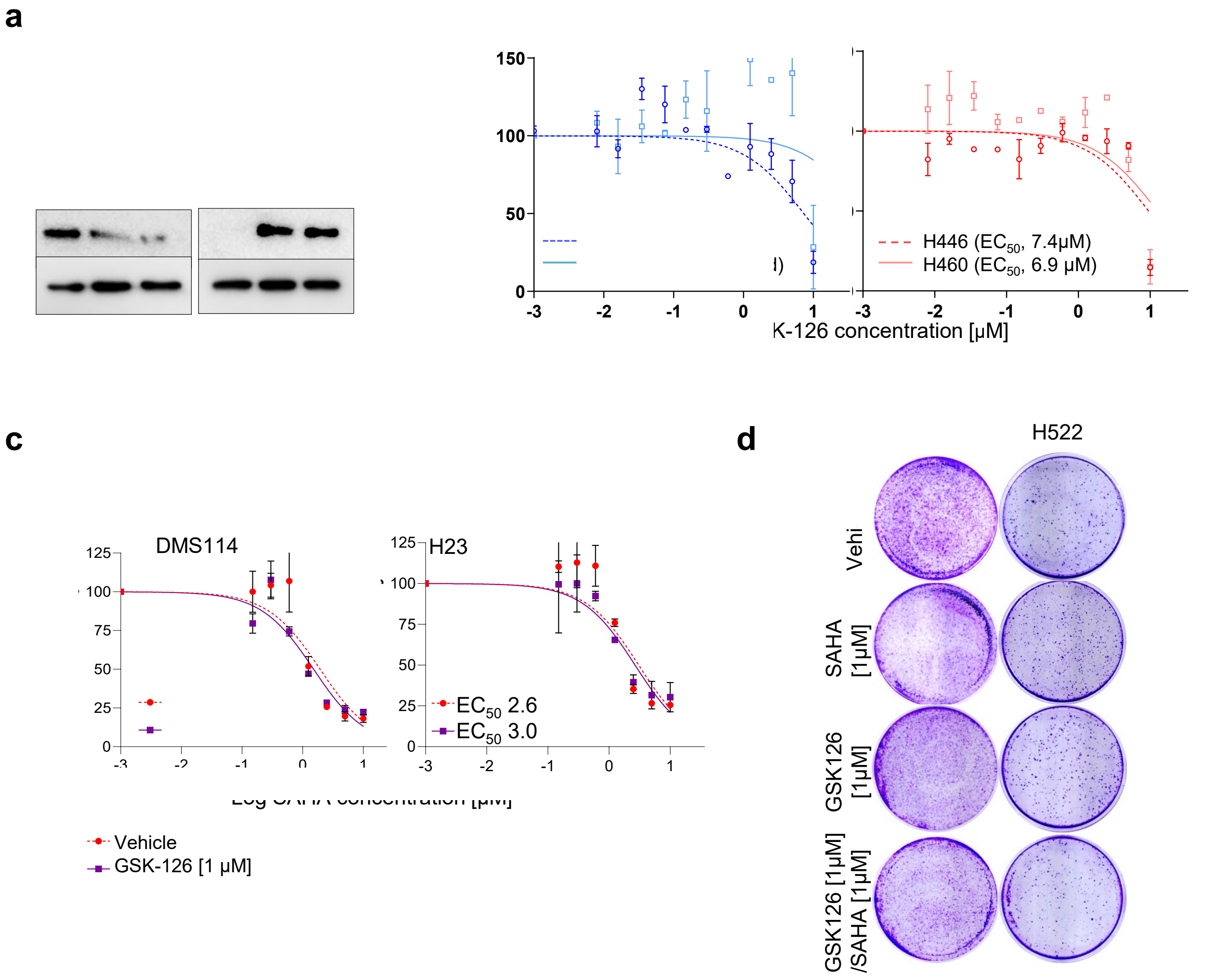

Supplementary Fig.3. GSK126, alone or in combination with SAHA, do not affect proliferation or viability in SMARCA4def cells a, Western blot depicting the levels of EZH2 in the indicated cancer cells, following SAHA treatment. ACTIN, protein-loading control. **b, c**, Effects on cell viability, measured using MTT assays, in the indicated cell lines after treatment with increasing concentrations of GSK-126 (**b**) or of SAHA and 1 μM of GSK-126, for 5 days (**c**). EC_{50}, half maximal effective concentration**.** Values from two independent experiments per cell line are represented**.** Error bars, means ± SD of three replicates**.** **, $P < 0.01$; ****, $P < 0.0001$. **d**, Representative clonogenic assays for the indicated cells and treatments. A Source Data file is available for this figure.

Supplementary Fig.4

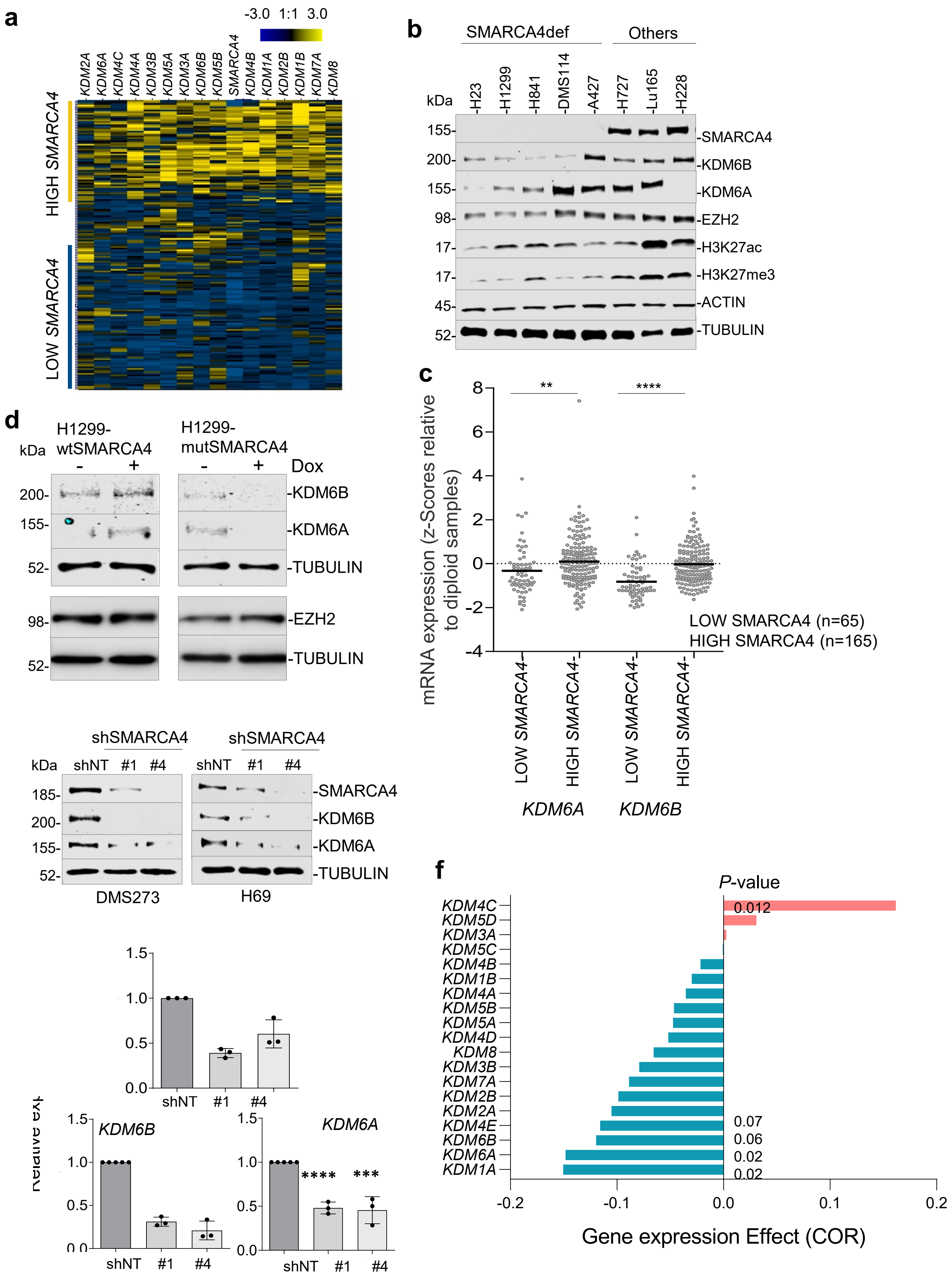

Supplementary Fig. 4. SMARCA4def cells carry lower levels of KDM6A and KDM6B, as compared to the MYCamp cells. a, Heatmap depicting mRNA levels (RPKMs, reads/kb/ million) of the indicated *KDMs* and of *SMARCA4* from a panel of 179 lung cancer cell lines (from Cancer Cell Line Encyclopedia-CCLE at cBioportal; https://www.cbioportal.org/). The heatmap was generated using the Software Genesis. **b,** Western blot depicting endogenous levels of the indicated proteins in lung cancer cell lines. ACTIN and TUBULIN, protein-loading controls. The H228 cells do not have KDM6A protein due to a genetic inactivating alteration at the *KDM6A* gene. **c,** Plot comparing the mRNA levels of the indicated *KDMs* of the SMARCA4-low expressing and SMARCA4-high expressing tumors (lung primary adenocarcinomas from TCGA, Nature 2014 (https://www.cbioportal.org/). Each dot represents a single tumor. Bars show mean ± SD. Two-sided unpaired Student's t-test. **, P =0.01; ****, $P < 0.0001$.**d,** Determination of protein levels of KDM6A, KDM6B and EZH2 by western blot in the H1299-wtSMARCA4 and H1299-mutSMARCA4 cell models (dox, doxycycline, 1 µg/mL; 72 h). Bars show mean ± SD. Two-sided unpaired Student's t-test. **e,** Upper panel, western blots depicting levels of indicated proteins in the indicated cells infected with the shNT and with different shSMARCA4 (#1, #4) (see Ref. 7 for more information). Lower panel, real-time quantitative PCR of *SMARCA4*, *KDM6A* and *KDM6B* (relative to ACTB) for comparing mRNA levels in the H69 cells infected with the shNT and with two different shSMARCA4 (#1, #4). n=3, for each gene and shRNA. Error bars, means ± SD of replicates**.** Two-sided unpaired Student's t-test. *, P =0.011; ***, P =0.002; ****, $P < 0.0001$. **f,** Analysis of the changes in the expression levels of the indicated transcripts from a panel of 242 genomically characterized human cancer cell lines after knockout of SMARCA4 using CRISPR/CAS9 (Sanger CRISPR). Data and statistics are available at the Depmap portal (https://depmap.org/portal/depmap/). A Source Data file is available for this figure.

Supplementary Fig.5

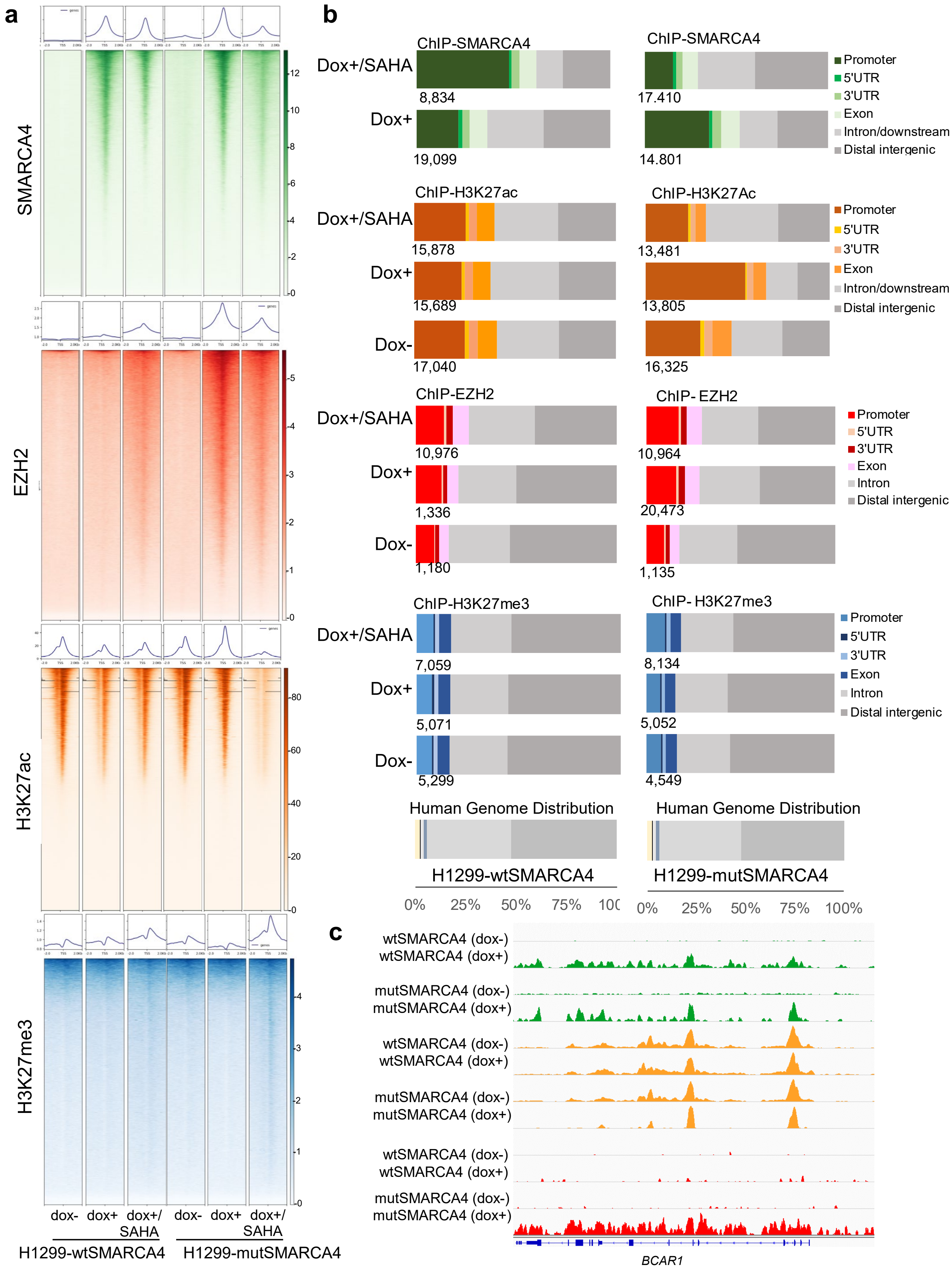

Supplementary Fig. 5. Genome-wide effects of wild type and mutant SMARCA4 and of SAHA on the dynamics of H3K27 modification and on EZH2 occupancy. a, Heatmaps of normalised ChIP-seq intensities, centred ± 2 kb around the transcriptional start sites (TSS), of SMARCA4, H3K27ac, H3K27me3 and EZH2, in the H1299 cell model. **b,** Genome-wide functional annotations for peaks generated by ChIP-seq analyses. Promoters are defined as the regions ± 2 kb around the annotated TSS. Numbers below each bar indicate the absolute number of peaks at promoters called for the ChIP-seq of each indicated protein and condition. **c,** Representative snapshots from IGV of ChIP-seq profiles at selected target loci, performed in the H1299 cell model. H1299-wtSMARCA4 or H1299-mutSMARCA4 cell models after induction of SMARCA4 (dox, doxycycline, 1 µg/mL; 72 h) with or without SAHA treatment (1 µM, 72 h).

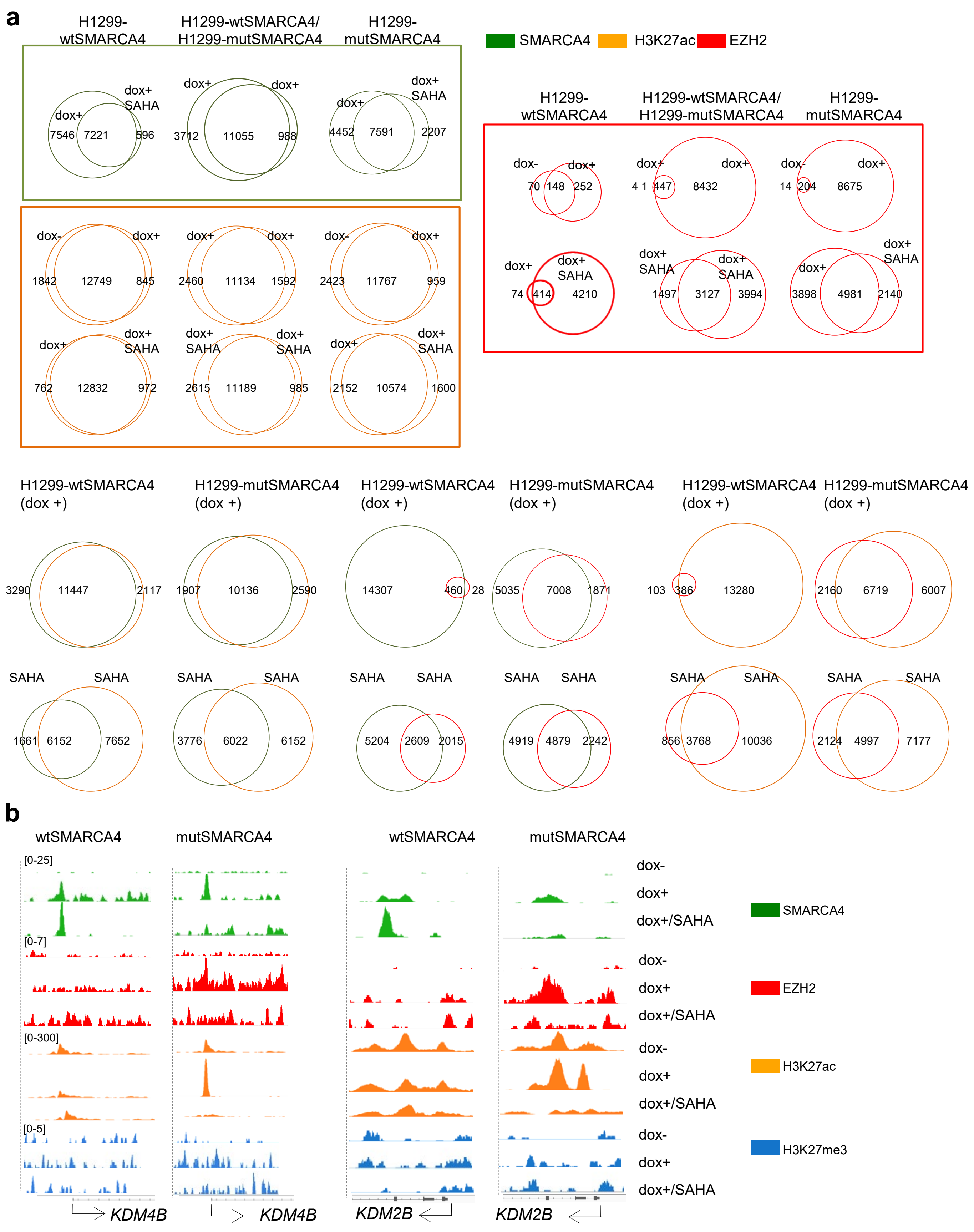
a
H1299-wtSMARCA4
H1299-wtSMARCA4/ H1299-mutSMARCA4
H1299-mutSMARCA4
SMARCA4
H3K27ac
EZH2
dox-
dox+
dox+ SAHA
H1299-wtSMARCA4 (dox +)
H1299-mutSMARCA4 (dox +)
SAHA
b
wtSMARCA4
mutSMARCA4
[0-25]
[0-7]
[0-300]
[0-5]
dox-
dox+
dox+/SAHA
SMARCA4
EZH2
H3K27ac
H3K27me3
KDM4B
KDM2B

Supplementary Fig. 6. Venn diagrams showing shared occupancies and examples of snapshots for the different ChIP-sequencing experiments. **a,** Venn diagrams of the overlap of SMARCA4, H3K27ac and EZH2 peaks in the H1299 cell model. **b,** Representative snapshots from IGV of ChIP-seq profiles at selected target loci, performed in the H1299 cell model. H1299-wtSMARCA4 or H1299-mutSMARCA4 cell models after induction of SMARCA4 (dox, doxycycline, 1 µg/mL; 72 h) with or without SAHA treatment (1 µM, 72 h).

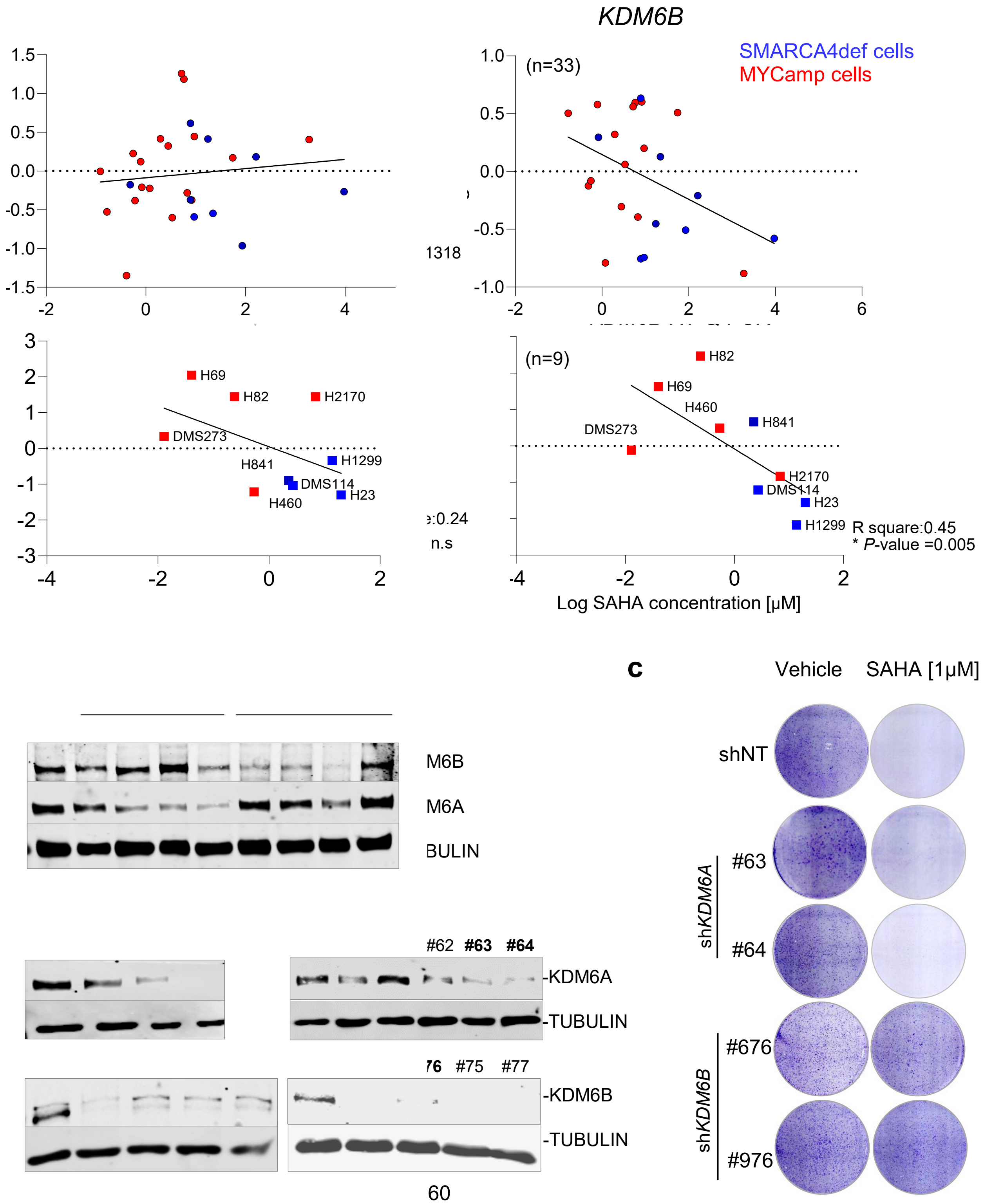
KDM6B
SMARCA4def cells
MYCamp cells
(n=33)
1318
(n=9)
H82
H69
H460
H841
DMS273
H2170
DMS114
H23
H1299
:0.24
n.s
R square:0.45
* P-value =0.005
Log SAHA concentration [μM]
M6B
M6A
BULIN
#62 #63 #64
-KDM6A
-TUBULIN
76 #75 #77
-KDM6B
-TUBULIN
c
Vehicle
SAHA [1μM]
shNT
shKDM6A
#63
#64
shKDM6B
#676
#976

Supplementary Fig. 7. Lower levels of KDM6A and KDM6B in the SMARCA4def cells and shRNAs, to downregulate KDM6A and KDM6B expression in different MYCamp cells. a, Significant correlation between mRNA levels of *KDM6B* (RNA-sequencing from the Sanger database or RT-QPCR data from our current study) and EC_{50} (half maximal effective concentration) for SAHA (upper panels, from www.cancerrxgene.org or lower panels, from our own data). Each dot represents a single cancer cell line**.** Simple linear regression analysis. *, $P < 0.05$; n.s, not significant. **b,** Western blots depicting levels of the indicated proteins in the indicated cells infected with the shNT and with different shKDM6A (#60, #61, #62, #63 and #64) and shKDM6B (#676, #976, #75 and #77). **c,** Clonogenic assays for the H460 cells infected with shNT, shKDM6A (#63, #64) or shKDM6B (#676, #976) treated with SAHA for 5 days. A Source Data file is available for this figure.

Supplementary Fig.8

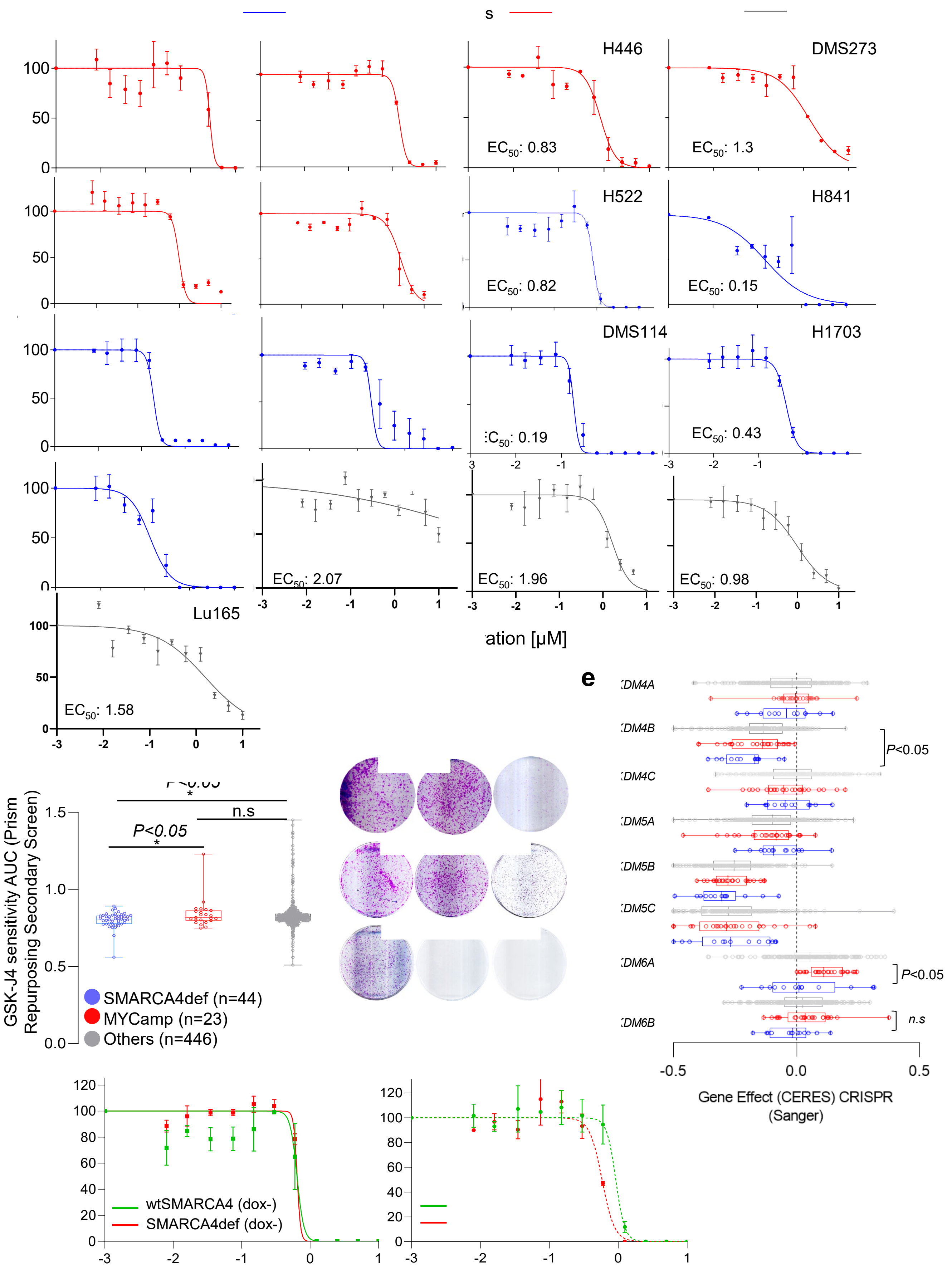

Supplementary Fig. 8. GSK-J4 induces cell growth inhibition of SMARCA4def lung cancer cells. a, Examples of the effects on cell viability, measured using MTT assays, after treatment with increasing concentrations of GSK-J4 in the indicated lung cancer cell lines. Lines, percentage of viable cells relative to the total number at 0 h. Error bars, means ± SD of three replicates. **b,** Analysis of the sensitivity to the GSK-J4 drug in a panel of 750 genomically characterized human cancer cell lines using the PRISM (Profiling Relative Inhibition Simultaneously in Mixtures) technology. Each dot represents a single cancer cell line. In box-whisker plots, the horizontal band inside box indicates the median, the bottom and top edges of the box 25th–75th percentiles and the whiskers indicate the min to max. Two-sided unpaired Student's t-test.*, P= 0.032 in both cases; n.s, not significant. Data available at the Achilles project at the depmap portal (https://depmap.org/portal/achilles/). **c-d,** clonogenic (**c**) and cell viability (MTT) (**d**) assays in the H1299-wtSMARCA4 or H1299-mutSMARCA4 cell models before and after induction of SMARCA4 (doxycycline, 1 µg/mL; 5 days) treated with GSK-J4. Lines, percentage of viable cells relative to the total number at 0 h. Error bars, mean ± SD of three replicates. **e,** Dependency of different cancer cell lines for each of the indicated KDM from the Achilles project at the depmap portal (https://depmap.org/portal/achilles/). Blue, SMARCA4def cell lines (n=13); Red, MYCamp cell lines (n=25) and grey (n=204), cancer cell lines that are wild type for both SMARCA4 and MYC. Two-sided unpaired Student's t-test. *, $P < 0.05$; n.s, not significant. In box-whisker plots, the horizontal band inside box indicates the median, the bottom and top edges of the box 25th–75th percentiles and the whiskers indicate the min to max. A Source Data file is available for this figure.

Supplementary Fig.9

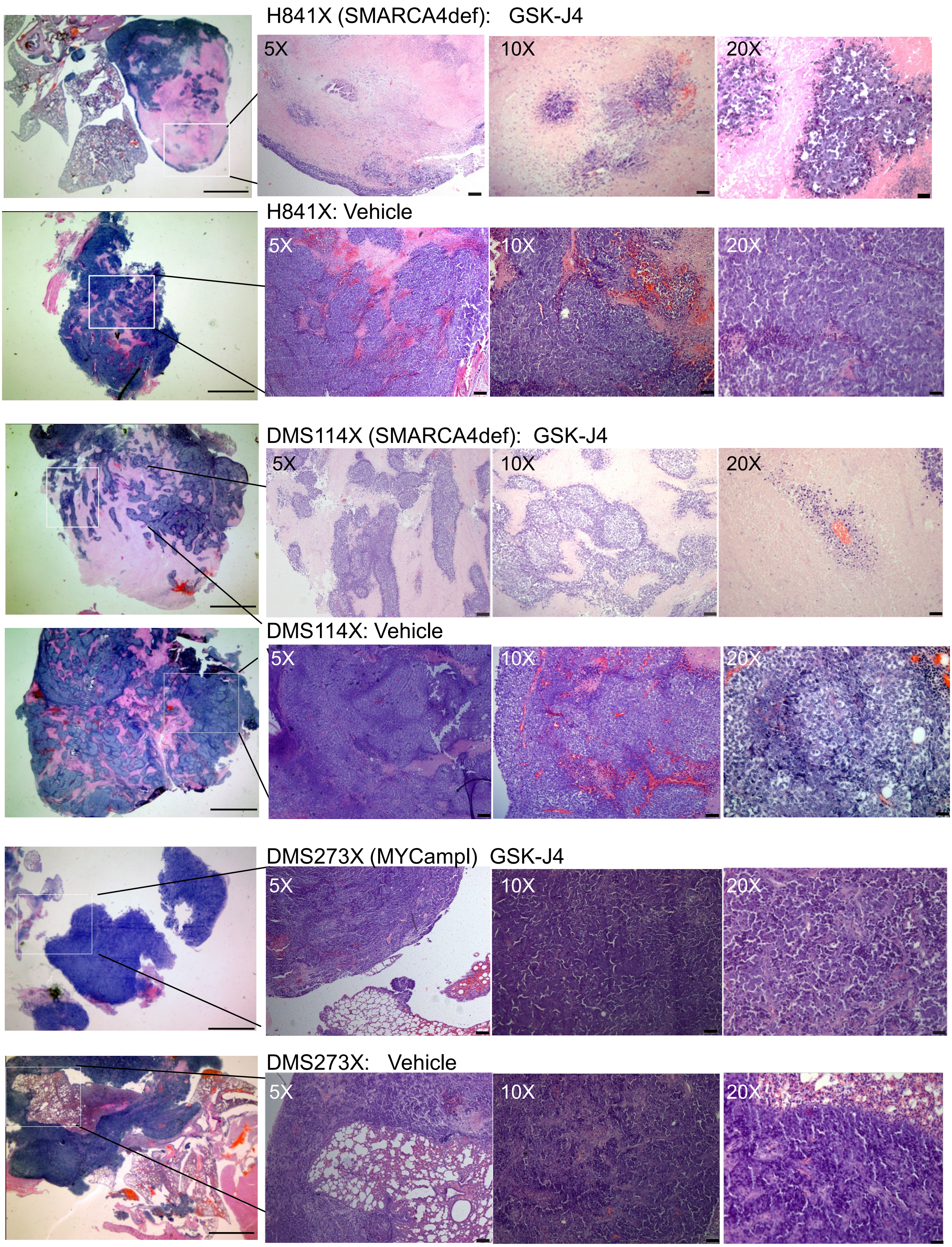

Supplementary Fig. 9. GSK-J4 induces tumour regression of SMARCA4def lung tumours *in vivo* Representative sections of H&E staining of the indicated tumours developed from the indicated lung cancer cells grown in nude mice (lung orthotopic implantation of small solid fragments of engrafted cell lines previously grown subcutaneously), treated with GSK-J4 or vehicle. Left panels, scale bars, 2.5 mm. Magnification as indicated (5x, 10x and 20x panels; scale bars, 100 µm, 50 µm and 25 µm, respectively).

Supplementary Fig.10

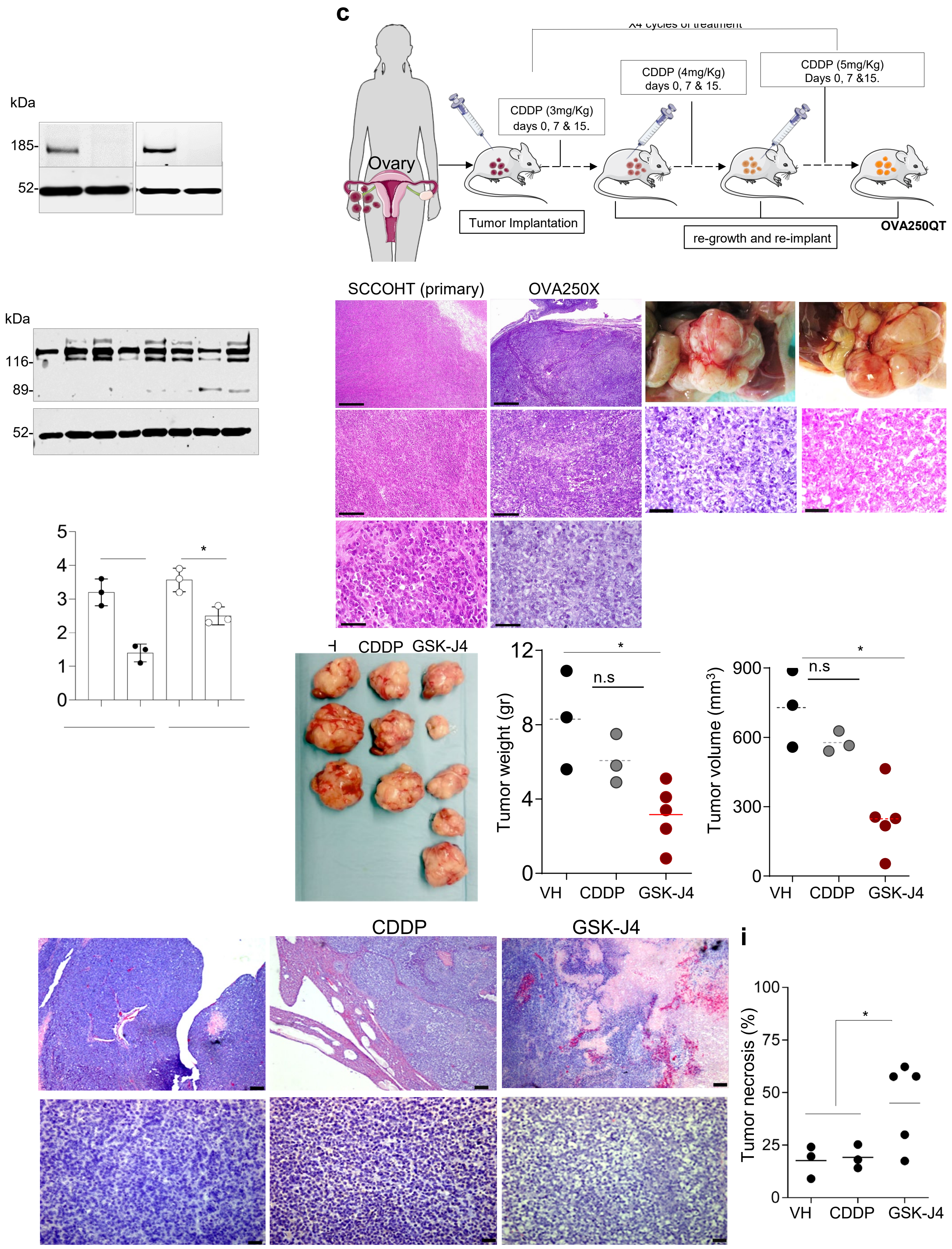

Supplementary Fig. 10. *In vivo* establishment of paired cisplatin-resistant SCCOHT xenografted tumour and effect of cisplatin and GSK-J4 treatments. a, Western blot to show presence or absence of SMARCA4 in the indicated ovarian cancer cells. **b**, Western blot depicting the cleavage of PARP in the indicated cells, following the different treatments or vehicle. TUBULIN, protein-loading control. **c**, The experimental approach used for cisplatin (CDDP)-resistant tumour generation combines: (i) iterative cycles of cisplatin treatment (3 doses of cisplatin administered by i.v. injection on days 0, 7 and 15); (ii) successive increase of administered doses through four cycles applied to different mice. Lateral laparotomy was conducted in isofluorane-anaesthetised mice. The ovary was mobilised and small tumour pieces of primary tumour were anchored on the mouse ovarian surface with prolene 7.0 sutures. Engrafted OVA250X tumours grew as large solid masses, usually to 1,000–1,500 mm^3 in diameter, which determined the time of sacrifice. Figure schematics were generated using https://smart.servier.com/ (https://creativecommons.org/licenses/by/3.0/). **d,** Representative H&E staining, from at least five different tumours of each group, reveals a similar morphology between primary SCCOHT and paired engrafted OVA250X. Top panels, scale bar 500 µm, middle panels scale bar, 200 µm. Bottom panels scale bar, 50 µm. **e,** Gross pathological photographs at necropsy (top panels) and representative H&E staining (bottom panels) of the OVA250X and OVA250XR tumours, from at least five different tumours of each group. Scale bar, 50 µm. **f,** Measure of the weight of each of the tumours from mice treated with vehicle (VH) (n=3) or cisplatin (CDDP) (n=3). *, P =0.046 (OVA250X) and *, P =0.018 (OVA250XR); Error bars, means ± SD of replicates**. g,** Left panel, gross pathological photographs of all ovarian tumours that developed in the mice treated with vehicle (VH) (n=3), cisplatin (CDP) (n=3) or GSK-J4 (n=5). Right panels, volume and weight of each tumour. *, P =0.036 in both cases; n.s, not significant. **h,** Representative H&E staining of OVA250XR tumours, from at least five different tumours of each group, treated as indicated. Scale bar, 100 µm (upper panels) and 25 µm (lower panels). **i**, Quantification of necrotic areas from the tumours from mice treated with vehicle (VH) (n=3) or cisplatin (CDDP) (n=3) or GSK-J4 (n=5). *, P =0.044; All tests are two-sided unpaired Student's t-testn.s, not significant. A Source Data file is available for this figure.

Supplementary Table 1. List of antibodies used. WB, western-blot; IHC, immunohistochemistry; ChIP, chromatin immunoprecipitation

Protein	Reference	Supplier	Technique	Dilution (WB)	Total amount (ChIP)	Dilution (IHC)
KDM6B/JMJD3	#3457	Cell signaling	WB	1:1000		
	ab38113	Abcam	WB & IHC	1:1000		1:50
KDM6A/UTX	#33510	Cell signaling	WB & IHC	1:1000		1:100
H3K27me3	07-449	Sigma	WB & ChIP-seq & IHC	1:1000	2µg	1:100
H3K27ac	ab4729	Abcam	WB & ChIP-seq & IHC	1:1000	2µg	1:100
EZH2	#5246	Cell signaling	WB & ChIP-seq	1:1000	2µg	
SMARCA4	#49360	Cell signaling	WB & ChIP-seq	1:1000	2µg	
ACTIN	A2228	Sigma	WB	1:10000		
TUBULIN	200-301-880S	Rockland	WB	1:10000		
PARP	#9542	Cell signaling	WB	1:1000		
CASPASE-3	#9668	Cell signaling	IHC	1:1000		1:100
Donkey anti-rabbit IgG IRDye 800CW fluorescent secondary antibody	926-32213	Li-Cor	WB	1:10000		
Donkey anti-mouse IgG IRDye 680LT fluorescent secondary antibody	926-68022	Li-Cor	WB	1:10000		

Supplementary Table 2. List of primers used for the quantitative RT-PCRs.

Gene	Forward (5'-3')	Reverse (5'-3')	Scale (µmols)	Supplier
ACTB	GGCATCCTCACCCTGAGGTA	AGGTGTGGTCGGAGATTTTC	0.025	Sigma
GUSB	CTGTACACGACACCCACCAC	GATGAGGAACTGGCTCTTGA	0.025	Sigma
KDM1A/LSD1	GCCATGGTGGTACAGGTCTA	TGGCCAGTTCCATATTTACA	0.025	Sigma
KDM2A	CGGATAGTTGAGAAAGCCAT	CTCTTTGGTGGGCCTCTGTA	0.025	Sigma
KDM3A	ACCTGCAGTTATTCTTCAGC	TAATGCCAGTCCTATGCCAT	0.025	Sigma
KDM4A/JMJD2A	CCTCACTGCGCTGTCTGTAT	CCAGTCGAAGTGAAGCACAT	0.025	Sigma
KDM4B/JMJD2B	GATCTCCATGGACGTGTTCG	CGTTTCCGGTGAGACCTGCC	0.025	Sigma
KDM4C/JMJD2C	GGCATAGGTGACAGGGTGTG	CGGGGACCAAACTCTGGAAA	0.025	Sigma
KDM4D/JMJD2D	CGGGATCTGCACAGATTATC	AGTTTCTGAGGAGGGCGACC	0.025	Sigma
KDM5A	GGTTTCTTAAGGTGGCAAGT	TTCTTTTGTACTGTTCCCTA	0.025	Sigma
KDM5B	AGCTTTTCTCAGAATGTTGG	GCAGAGTCTGGGAATTCACA	0.025	Sigma
KDM5C	GGGTTTCTAAAGTGTAGATC	CCCACACATCTGAGCTTTAG	0.025	Sigma
KDM6A/UTX	GTCCGAGTGTCAACCAACTG	TGAGAGTCCTGGAGTAGGAG	0.025	Sigma
KDM6B/JMJD3	CTCAACTTGGGCCTCTTCTC	GCCTGTCAGATCCCAGTTCT	0.025	Sigma

Supplementary Table 3. References for the shRNAs

Name	Clone ID	Vector	Target gene	Supplier
#60	TRCN0000107760	pLKO.1	*KDM6A*	Sigma
#61	TRCN0000107761	pLKO.1	*KDM6A*	Sigma
#63	TRCN0000107763	pLKO.1	*KDM6A*	Sigma
#64	TRCN0000107764	pLKO.1	*KDM6A*	Sigma
#75	TRCN0000236675	pLKO.1	*KDM6B*	Sigma
#77	TRCN0000236677	pLKO.1	*KDM6B*	Sigma
#676	TRCN0000236676	pLKO.1	*KDM6B*	Sigma
#976	TRCN0000359976	pLKO.1	*KDM6B*	Sigma

Supplementary Table 4. List of primers used for the cloning of KDM6A and KDM6B.

Gene	Forward (5'-3')	Reverse (5'-3')	Scale (µmols)	Supplier
KDM6A	aaaaaaaaCTCGAGCCGCCACCATGAAATCCTGCGGAGTGTCGCTCGCTAC	aaaaaaaaGCGGCCGCTCAAGATGAGGCGGATGGTAATGGAGGAGCTAATG	0.025	Sigma
KDM6B	ttttttttGGTACCGCCGCCACCATGCATCGGGCAGTGGACCCTCCAG	ttttttttCTCGAGTCATCGCGACGTGCTGGCTGGGG	0.025	Sigma

Supplementary Table 5. List of primers used for the sequencing of KDM6A and KDM6B.

Gene	Forward (5'-3')	Scale (µmols)	Supplier
KDM6A	ATGAGTCTAGTTTAAAGCATTTTCAG	0.025	Sigma
KDM6A	ATTAAATGCTACTTAAATGCAACTAG	0.025	Sigma
KDM6A	GGAGTTGCACAGGTAC	0.025	Sigma
KDM6A	GGTGGACAACAAGGC	0.025	Sigma
KDM6A	TAAAAATGGCTTATCTAACAGTAGC	0.025	Sigma
KDM6A	TGACTAAACTTCCTGCTTTTG	0.025	Sigma
KDM6A	GCACGAAATATCAAGGTCTCAGATCCAAAGC	0.025	Sigma
KDM6B	AACGGAACTATGGAGCC	0.025	Sigma
KDM6B	CTCCAGCGTTTCACC	0.025	Sigma
KDM6B	AGGATTCTCACACCCC	0.025	Sigma
KDM6B	CCGCTGAAGGAGCCC	0.025	Sigma
KDM6B	CGAAGAGCCAGTCCC	0.025	Sigma
KDM6B	TGCCCAGCCCACACC	0.025	Sigma
KDM6B	GTGGAAGCGAGTGGC	0.025	Sigma
KDM6B	ACGAGCACTACTGGG	0.025	Sigma
KDM6B	AAGAAAATCGCTTACCAGGGC	0.025	Sigma

Supplementary Table 6. Distribution of H3K27me3 staining among tumours (three replicates per cell line and condition) from the DMS273X, H841X, DMS114X and OVA250X tumours treated with vehicle or GSK-J4. Low (intensity values 1 & 2); high (values 3 & 4).

		Eval 1		Eval 2		Eval 3		Mean		Categories	
		H3K27me3		H3K27me3		H3K27me3		H3K27me3		H3K27me3	
	Replicate	Vehicle	GSK-J4	Vehicle	GSK-J4	Vehicle	GSK-J4	Mean-Vehicle	Mean-GSK-J4	Categories Vehicles	Categories GSK-J4
**DMS114X**	R1	1	3	1	2	1	1	1	2	Low	Low
	R2	2	4	2	3	2	3	2	3.3	Low	High
	R3	2	4	2	4	2	4	2	4	Low	High
**H841X**	R1	1	3	1	3	1	3	1	3	Low	High
	R2	3	4	3	3	3	3	3	3.3	High	High
	R3	2	4	2	4	2	3	2	3.6	Low	High
**DMS273X**	R1	2	3	1	2	1	2	1.3	2.3	Low	Low
	R2	1	3	2	3	2	3	1.6	3	Low	High
	R3	2	4	2	3	2	4	2	3.6	Low	High
**OVA250X**	R1	2	3	1	3	2	3	1.6	3	Low	High
	R2	2	4	1	4	2	4	1.6	4	Low	High
	R3	2	3	2	4	3	4	2.3	3.6	Low	High
