# Supplementary material for: SMARCA4 deficient tumours are vulnerable to KDM6A/UTX and KDM6B/JMJD3 blockade
Source: Nat Commun. 2021 Jul 14;12:4319. doi: 10.1038/s41467-021-24618-3 (PMC8280185; doi:10.1038/s41467-021-24618-3)
Supplement: Supplementary file 3 — Reporting Summary [file 41467_2021_24618_MOESM3_ESM.pdf]

nature research

Corresponding author(s): Sanchez-Cespedes M. and Romero OA.

Last updated by author(s): Jun 1, 2021

Reporting Summary

Nature Research wishes to improve the reproducibility of the work that we publish. This form provides structure for consistency and transparency in reporting. For further information on Nature Research policies, see Authors & Referees and the Editorial Policy Checklist.

Statistics

For all statistical analyses, confirm that the following items are present in the figure legend, table legend, main text, or Methods section.

n/a	Confirmed	
☐	☒	The exact sample size (*n*) for each experimental group/condition, given as a discrete number and unit of measurement
☐	☒	A statement on whether measurements were taken from distinct samples or whether the same sample was measured repeatedly
☐	☒	The statistical test(s) used AND whether they are one- or two-sided<br>*Only common tests should be described solely by name; describe more complex techniques in the Methods section.*
☐	☒	A description of all covariates tested
☒	☐	A description of any assumptions or corrections, such as tests of normality and adjustment for multiple comparisons
☐	☒	A full description of the statistical parameters including central tendency (e.g. means) or other basic estimates (e.g. regression coefficient) AND variation (e.g. standard deviation) or associated estimates of uncertainty (e.g. confidence intervals)
☐	☒	For null hypothesis testing, the test statistic (e.g. *F*, *t*, *r*) with confidence intervals, effect sizes, degrees of freedom and *P* value noted<br>*Give P values as exact values whenever suitable.*
☒	☐	For Bayesian analysis, information on the choice of priors and Markov chain Monte Carlo settings
☒	☐	For hierarchical and complex designs, identification of the appropriate level for tests and full reporting of outcomes
☐	☒	Estimates of effect sizes (e.g. Cohen's *d*, Pearson's *r*), indicating how they were calculated

Our web collection on statistics for biologists contains articles on many of the points above.

Software and code

Policy information about availability of computer code

Data collection	Illumina Hiseq2500 instrument and manufacturer provided software was used to collect ChIP-seq data. Leica DM1000 microscope was used to evaluate histopatological samples and tissue sections. qRT-PCR was performed in a Quantstudio 5 Real-Time PCR Instrument. Odyssey Li-Cor CLx was used for Western Blot image acquisition. Flow cytometry experiments for detecting PI emission were performed in BD FACSCanto II Cell Analyzer.
Data analysis	Commercial and publicly available softwares were used to analyze all data in this study. These include Microsoft Excel 2016, Adobe Photoshop 2021, Image Studio Lite v5.2, Quantstudio Design & Analysis v1.5.1, Prism GraphPad 9, FACSDiva v6.1.2, FlowJo v7.6, DeepTools v3.5, Bowtie v1.2.2, R package CHIPpealAnno v3.15, MACS2 v2.1.1, HiSeq Control Software (HCS) 2.2.68, PICARD Tools, Compusyn software, Homer software, Agilent feature extraction software and Genesis software.

For manuscripts utilizing custom algorithms or software that are central to the research but not yet described in published literature, software must be made available to editors/reviewers. We strongly encourage code deposition in a community repository (e.g. GitHub). See the Nature Research guidelines for submitting code & software for further information.

Data

Policy information about availability of data

All manuscripts must include a data availability statement. This statement should provide the following information, where applicable:

- Accession codes, unique identifiers, or web links for publicly available datasets
- A list of figures that have associated raw data
- A description of any restrictions on data availability

The ChIP-seq raw data obtained in this study has been uploaded to the Gene Expression Omnibus-GEO (NCBI), under accession number GSE155129 (https://www.ncbi.nlm.nih.gov/geo/query/acc.cgi?acc=GSE155129). Databases/Datasets used in the study were the following: cBioPortal for Cancer Genomics (https://www.cbioportal.org/), COSMIC-Catalogue of Somatic Mutations in Cancer (https://cancer.sanger.ac.uk/cell_lines), DepMap Portal-Cancer Dependency Map (https://depmap.org/portal/depmap/), Genomics of Drug Sensitivity in Cancer database (www.cancerrxgene.org). Cancer Cell Line Encyclopedia-CCLE (https://

portals.broadinstitute.org/ccle). The authors declare that other data supporting the findings of this study are provided in the Supplementary Information/Source Data file. Uncropped western blot images are also present in a Source Data file. Source data are provided with this paper.

Field-specific reporting

Please select the one below that is the best fit for your research. If you are not sure, read the appropriate sections before making your selection.

☒ Life sciences ☐ Behavioural & social sciences ☐ Ecological, evolutionary & environmental sciences

For a reference copy of the document with all sections, see nature.com/documents/nr-reporting-summary-flat.pdf

Life sciences study design

All studies must disclose on these points even when the disclosure is negative.

Sample size	Experiments were designed to have enough sample sizes to obtain reliable results. For in vitro cell experiments, we used all the cell lines at our disposal for each genetic alteration relative to MYC amplification, SMARCA4 inactivation or other mutations with a minimum number of 3. At least three independent replicates and a minimum of two biological replicates were used for each experiment to ensure the reproducibility and to perform statistical analysis. For in vivo experiments, we chose a higher number of animals in the treated group compared with the control group in order to homogenize potential toxic side effects of the drug. In vivo sample sizes were based on previous studies in the laboratory that revealed that this number of animals to be enough to obtain significant differences. Student's t-tests, EC50 calculations, Kaplan-Meier estimates and log-rank (Mantel-Cox) test were performed using Prism software (GraphPad Prism 9).
Data exclusions	No data were excluded from analysis.
Replication	Data was obtained from three technical replicates and in at least two biological replicates with successful outcomes. Independent biologic replicates are shown in all figures.
Randomization	Cells were allocated into experimental groups based on its genetical alteration (MYCamp, SMARCA4def or Others). In all genetic based groups the experiments includ the control sample and treated onesfor each cancer cell line in each group Mice were randomly divided into control and experimental groups at the start of each experiment. They were also randomized and intraperitoneally treated with GSK-J4 or corresponding vehicle only.
Blinding	In vitro experiments were not blinded in order to allow the investigators to a correct identitfication of samples and to ensure then a correct data collection. In other hand, blinding strategy was used specifically in the IHC valoration.

Reporting for specific materials, systems and methods

We require information from authors about some types of materials, experimental systems and methods used in many studies. Here, indicate whether each material, system or method listed is relevant to your study. If you are not sure if a list item applies to your research, read the appropriate section before selecting a response.

Materials & experimental systems

n/a	Involved in the study	
☐	☒	Antibodies
☐	☒	Eukaryotic cell lines
☒	☐	Palaeontology
☐	☒	Animals and other organisms
☐	☒	Human research participants
☒	☐	Clinical data

Methods

n/a	Involved in the study	
☐	☒	ChIP-seq
☐	☒	Flow cytometry
☒	☐	MRI-based neuroimaging

Antibodies

Antibodies used

All antibodies used in this study were obtained from commercial suppliers (Sigma Aldrich, Cell Signaling, Rockland, Sigma Aldrich, Li-Cor and Abcam). A list of relevant information on the antibodies is provided below:
Anti-Rabbit JMJD3 (KDM6B), Cell Signaling 3457S, Polyclonal, Lot 3, 1:1000
Anti-Rabbit JMJD3 (KDM6B), Abcam ab38113, Polyclonal, Lot GR3228142-1, 1:1000
Anti-Rabbit EZH2, Cell Signaling 5246S, Clone D2C9, Lot 7 , 1:1000
Anti-Rabbit UTX (KDM6A), Cell Signaling 33510S, Clone D3Q1l, Lot 3 , 1:1000
Anti-Rabbit BRG1 (SMARCA4), Cell Signaling 49360S, Clone D1Q7F, Lot 1, 1:1000
Anti-Rabbit H3K27me3, Sigma Aldrich 07-449, Polyclonal, Lot 2919706, 1:1000
Anti-Rabbit H3K27ac, Abcam ab4729, Polyclonal, Lot GR3216173-1, 1:1000
Anti-Rabbit PARP, Cell Signaling 9542S, Polyclonal, Lot 3, 1:1000
Anti-Rabbit CASPASE3, Cell Signaling 9668S, Clone 3G2, Lot 7, 1:1000
Anti-Mouse alpha-Tubulin, Rockland 200-301-880S, Clone 17H11.F10, Lot 32803 ,1:10000
Anti-Mouse beta-Actin, Sigma Aldrich A2228, Clone AC-74, Lot 2918908, 1:10000

Donkey anti-rabbit IgG IRDye 800CW fluorescent secondary antibody, Li-Cor 926-32213, Lot C90129-01, 1:10000
Donkey anti-mouse IgG IRDye 680LT flourescent secondary antibody, Li-Cor 926-68022, Lot C90821-12, 1:10000

Validation

All the antibodies used in the study were bought from commercial vendors and were validated by the manufacturers, in other studies and/or in the current study:

BRG1 (49360S). validation for the BRG1 antibody was performed in this study using shRNAs to deplete BRG1 (SMARCA4) expression in cancer cells and an inducible model of wild type and mutant version of SMARCA4. SMARCA4 deficient cells were included as negative controls whereas cells with MYC overexpression were considered positive controls.

JMJD3 (3457S); UTX (33510S). validations for KDM6A (UTX) and KDM6B (JMJD3) antibodies were performed in the current study, as follows: KDM6A and KDM6B knock down (shRNA) cancer cells were included as negative controls for the KDM6A and KDM6B antibodies, respectively, whereas cells with MYC overexpression, by gene amplification, were considered positive controls.

JMJD3 (ab38113). WB and IHC validation: Xun J, Wang D, Shen L, Gong J, Gao R, Du L, Chang A, Song X, Xiang R, Tan X. JMJD3 suppresses stem cell-like characteristics in breast cancer cells by downregulation of Oct4 independently of its demethylase activity. Oncotarget. 2017 Mar 28;8(13):21918-21929. doi: 10.18632/oncotarget.15747. PMID: 28423536; PMCID: PMC5400634.

EZH2(5246S).WB and ChIP validation: Ye M, Xie L, Zhang J, et al. Determination of long non-coding RNAs associated with EZH2 in neuroblastoma by RIP-seq, RNA-seq and ChIP-seq. Oncol Lett. 2020;20(4):1. doi:10.3892/ol.2020.11862

H3K27me3 (07-449).
ChIP validation: Brinkman AB, Gu H, Bartels SJ, et al. Sequential ChIP-bisulfite sequencing enables direct genome-scale investigation of chromatin and DNA methylation cross-talk. Genome Res. 2012;22(6):1128-1138. doi:10.1101/gr.133728.111.
IHC validation: Abe M, Tsai SY, Jin SG, Pfeifer GP, Szabó PE. Sex-specific dynamics of global chromatin changes in fetal mouse germ cells. PLoS One. 2011;6(8):e23848. doi:10.1371/journal.pone.0023848.
WB validation: Kim W, Bird GH, Neff T, et al. Targeted disruption of the EZH2-EED complex inhibits EZH2-dependent cancer. Nat Chem Biol. 2013;9(10):643-650. doi:10.1038/nchembio.1331

H3K27ac (ab4729)
WB and ChIP validation: Martinez Calejman C, Trefely S, Entwisle SW, Luciano A, Jung SM, Hsiao W, Torres A, Hung CM, Li H, Snyder NW, Villén J, Wellen KE, Guertin DA. mTORC2-AKT signaling to ATP-citrate lyase drives brown adipogenesis and de novo lipogenesis. Nat Commun. 2020 Jan 29;11(1):575. doi: 10.1038/s41467-020-14430-w. Erratum in: Nat Commun. 2020 Sep 8;11(1):4585. PMID: 31996678; PMCID: PMC6989638.
IHC validation: Tao H, Li Q, Lin Y, et al. Coordinated expression of p300 and HDAC3 upregulates histone acetylation during dentinogenesis. J Cell Biochem. 2020;121(3):2478-2488. doi:10.1002/jcb.29470

PARP (9542S). WB validation: Latif AL, Newcombe A, Li S, Gilroy K, Robertson NA, Lei X, Stewart HJS, Cole J, Terradas MT, Rishi L, McGarry L, McKeeve C, Reid C, Clark W, Campos J, Kirschner K, Davis A, Lopez J, Sakamaki JI, Morton JP, Ryan KM, Tait SWG, Abraham SA, Holyoake T, Higgins B, Huang X, Blyth K, Copland M, Chevassut TJT, Keeshan K, Adams PD. BRD4-mediated repression of p53 is a target for combination therapy in AML. Nat Commun. 2021 Jan 11;12(1):241. doi: 10.1038/s41467-020-20378-8. PMID: 33431824; PMCID: PMC7801601.

CASPASE-3 (9668S). WB validation: Lai SW, Bamodu OA, Chen JH, Wu AT, Lee WH, Chao TY, Yeh CT. Targeted PARP Inhibition Combined with FGFR1 Blockade is Synthetically Lethal to Malignant Cells in Patients with Pancreatic Cancer. Cells. 2020 Apr 8;9(4):911. doi: 10.3390/cells9040911. PMID: 32276472; PMCID: PMC7226837.

Alpha-Tubulin (200-301-880S). WB validation: Nguyen T, Kirsch BJ, Asaka R, Nabi K, Quinones A, Tan J, Antonio MJ, Camelo F, Li T, Nguyen S, Hoang G, Nguyen K, Udupa S, Sazeides C, Shen YA, Elgogary A, Reyes J, Zhao L, Kleensang A, Chaichana KL, Hartung T, Betenbaugh MJ, Marie SK, Jung JG, Wang TL, Gabrielson E, Le A. Uncovering the Role of N-Acetyl-Aspartyl-Glutamate as a Glutamate Reservoir in Cancer. Cell Rep. 2019 Apr 9;27(2):491-501.e6. doi: 10.1016/j.celrep.2019.03.036. PMID: 30970252; PMCID: PMC6472703.

Beta-Actin (A2228). WB validation: Bunnell TM, Burbach BJ, Shimizu Y, Ervasti JM. β-Actin specifically controls cell growth, migration, and the G-actin pool. Mol Biol Cell. 2011 Nov;22(21):4047-58. doi: 10.1091/mbc.E11-06-0582. Epub 2011 Sep 7. PMID: 21900491; PMCID: PMC3204067.

Eukaryotic cell lines

Policy information about cell lines

Cell line source(s)

The cell lines NCI-H841, NCI-H23, NCI-H82, NCI-H69, NCI-H157, NCI-H1975, NCI-H2170, NCI-H460, NCI-H1963, NCI-H446, DMS114, NCI-H727, NCI-H228, NCI-H128 and NCI-H1299 cell lines are from the American Type Culture Collection (ATCC). The DMS273 cell line is from the European Collection of Authenticated Cell Cultures (ECACC). The Lu165 cells were obtained from the RIKEN Cell Bank (Japan). One primary lung cancer cell line PCD11 was derived from malignant pleural effusions. Two primary cancer cell lines cultures were derived from orthoxenografts/PDOXs generated in nude mice from two primary tumours of two SCCOHT patients (OVA250L and OVA259L). Single cells and clumps were transferred to cell culture plates and maintained in DMEM supplemented with 10% FBS plus 50 U/mL penicillin and 50 mg/mL streptomycin under standard culture conditions. When cell colonies with epithelial cell morphology were observed, cells were trypsinized and expanded. Both primary cell lines were considered established after > 6 passages in vitro.

Authentication

Cell lines were not authenticated using STR profiling, Karyotyping or DNA barcoding. Cell lines were authenticated in our lab via PCR and direct sequencing of known oncogenes or tumors suppressor genes (e.g. TP53, SMARCA4), high level expression of the MYC-family of genes and routine observation of cell morphology under the microscope.

Mycoplasma contamination	All cell lines were tested for mycoplasma contamination in our institute. All cell lines had a negative result for Mycoplasma contamination.
Commonly misidentified lines (See ICLAC register)	A commonly misidentified cell line was used in the study is NCI-H157. However, we previously validated its genetic inactivation in SMARCA4.

Animals and other organisms

Policy information about studies involving animals; ARRIVE guidelines recommended for reporting animal research

Laboratory animals	Male and female athymic nu/nu mice (ENVIGO) 4-6 weeks old
Wild animals	No wild animals were used in the study
Field-collected samples	No field collected samples were used in the study
Ethics oversight	All animal experiments were approved by the IDIBELL Ethical Committee under protocol 9111 approved by the Government of Catalonian, AAALAC accredited Unit 1155, and performed in accordance with guidelines stated in the International Guiding Principles for Biomedical Research Involving Animals, developed by the Council for International Organizations of Medical Sciences (CIOMS).PDOXs generated in nude mice from two primary tumours of two SCCOHT patients (OVA250and OVA259) that were obtained from Bellvitge Hospital and the Catalan Institute of Oncology (ICO) with the approval of the Ethical Committee (CEIC Bellvitge). Ethical and legal protection guidelines of human subjects, including informed consent, were followed. Fresh orthoxenografts/PDOXs grown in the mouse ovaries were collected when mice were sacrificed at passage #1

Note that full information on the approval of the study protocol must also be provided in the manuscript.

Human research participants

Policy information about studies involving human research participants

Population characteristics	Human samples are primary tumours derived from SCCOHT patients (OVA250L and OVA259L), two women of 19 and 38 years with validated SMARCA4 inactivation, that were obtained from Bellvitge Hospital and the Catalan Institute of Oncology (ICO).
Recruitment	SCCOHT patients were recruited in the Hospital with an informed consent. Tumour samples were obtained as a left overs from surgery.
Ethics oversight	Samples were collected in accordance with the approval of the Ethical Committee (CEIC Bellvitge). Ethical and legal protection guidelines of human subjects were followed.

Note that full information on the approval of the study protocol must also be provided in the manuscript.

ChIP-seq

Data deposition

☒ Confirm that both raw and final processed data have been deposited in a public database such as GEO.

☒ Confirm that you have deposited or provided access to graph files (e.g. BED files) for the called peaks.

Data access links *May remain private before publication.*	The ChIP-seq data obtained in this study has been uploaded to the Gene Expression Omnibus-GEO (NCBI), under accession number GSE155129
Files in database submission	I1_23979_TCGAAC.fastq.gz<br>I2_23980_TCCTCT.fastq.gz<br>I3_23981_CCTGCT.fastq.gz<br>I4_23982_TGCGTC.fastq.gz<br>I5_23983_GTCAAC.fastq.gz<br>I6_23984_GTTCAG.fastq.gz<br>B6_23966_CTGATA.fastq.gz<br>B5_23965_GAACTC.fastq.gz<br>B4_23964_GCAACT.fastq.gz<br>B3_23963_CCGCAA.fastq.gz<br>B2_23962_ATGAGA.fastq.gz<br>B1_23961_CAAGGA.fastq.gz<br>E1_23955_CATTCG.fastq.gz<br>E2_23956_GCTCCA.fastq.gz<br>E3_23957_CCGGTT.fastq.gz<br>E4_23958_AATGCA.fastq.gz<br>E5_23959_AGTTGC.fastq.gz<br>E6_23960_TGACTT.fastq.gz<br>HA1_23967_GTTCGG.fastq.gz<br>HA2_23968_AACCGG.fastq.gz

HA3_23969_CATAAC.fastq.gz
HA4_23970_TAAGAT.fastq.gz
HA5_23971_TGGATT.fastq.gz
HA6_23972_AACGGT.fastq.gz
HM1_23973_GGCCTC.fastq.gz
HM2_23974_TGACCA.fastq.gz
HM3_23975_TTCAAG.fastq.gz
HM4_23976_GACTAA.fastq.gz
HM5_23977_ATAATT.fastq.gz
HM6_23978_TCAGAT.fastq.gz
B6_23966_CTGATA_REP2.fastq.gz
B5_23965_GAACTC_REP2.fastq.gz
B4_23964_GCAACT_REP2.fastq.gz
B3_23963_CCGCAA_REP2.fastq.gz
E1_23955_CATTCG_REP2.fastq.gz
E2_23956_GCTCCA_REP2.fastq.gz
E3_23957_CCGGTT_REP2.fastq.gz
E4_23958_AATGCA_REP2.fastq.gz
E5_23959_AGTTGC_REP2.fastq.gz
E6_23960_TGACTT_REP2.fastq.gz
HA1_23967_GTTCGG_REP2.fastq.gz
HA2_23968_AACCGG_REP2.fastq.gz
HA3_23969_CATAAC_REP2.fastq.gz
HA4_23970_TAAGAT_REP2.fastq.gz
HA5_23971_TGGATT_REP2.fastq.gz
HA6_23972_AACGGT_REP2.fastq.gz
HM1_23973_GGCCTC_REP2.fastq.gz
HM2_23974_TGACCA_REP2.fastq.gz
HM3_23975_TTCAAG_REP2.fastq.gz
HM4_23976_GACTAA_REP2.fastq.gz
HM5_23977_ATAATT_REP2.fastq.gz
HM6_23978_TCAGAT_REP2.fastq.gz

Genome browser session
(e.g. UCSC)

No longer applicable

Methodology

Replicates

Two technical replicates for each cell lines were used in this study.

Sequencing depth

M1 15846391
M2 15305989
M3 18851717
M4 18468872
E1 18623997
E2 18643765
E3 15810595
E4 14326104
E5 15293283
E6 15340860
B1 15303214
B2 9425042
B3 10134251
B4 12272919
B5 18046955
B6 17894228
HA1 14116670
HA2 14608789
HA3 15136015
HA4 14083992
HA5 15873453
HA6 14704232
HM1 14990751
HM2 13983073
HM3 19089013
HM4 16401884
HM5 17371040
HM6 18712031
I1 7741232
I2 7997517
I3 9324399
I4 12194530
I5 10176824
I6 13525421

M1_REP2 13761463
M2_REP2 14133133
M3_REP2 23671429
M4_REP2 23551658
E1_REP2 23154033
E2_REP2 23682075
E3_REP2 19954042
E4_REP2 17662035
E5_REP2 19075170
E6_REP2 14336772
B3_REP2 9818339
B4_REP2 11791798
B5_REP2 22697929
B6_REP2 23049515
HA1_REP2 17254930
HA2_REP2 11412023
HA3_REP2 12608631
HA4_REP2 12984591
HA5_REP2 12599312
HA6_REP2 11726276
HM1_REP2 24033960
HM2_REP2 22743857
HM3_REP2 31689502
HM4_REP2 27231270
HM5_REP2 34531551
HM6_REP2 33398640

Antibodies

anti-SMARCA4 49360S (1:1000, Cell Signaling Technology); anti-EZH2 5246S (1:1000, Cell Signaling); anti-H3K27ac ab4729 (1:1000, Abcam) anti-H3K27me3 07-449 (1:1000, Sigma Aldrich)

Peak calling parameters

Peaks were called using MACS2 v2.1.136.To avoid false positives, peaks were discarded if they were present in the ChIP-seq ofSMARCA4 in the SMARCA4-deficient cells. Genomic peak annotation was performed withthe R package ChIPpeakAnno v3.15, considering the region ± 2 kb around the TSS as the promoter. All analyses considered peaks overlapping with promoter regions, unless otherwisespecified. Peak lists were then transformed to gene target lists.

Data quality

For ChIP-sequencing data analysis, reads were aligned to the human reference genome hg38, using Bowtie v1.2.2, with default parameters and disallowing multi-mapping (–m 1)35.PCR duplicates were removed using PICARD (http://broadinstitute.github.io/picard/).Ambiguous and multi-mapped reads were discarded.

Software

PICARD (http://broadinstitute.github.io/picard/), MACS2 v2.1.1, Bowtie v1.2.2 and R package ChIPpeakAnno v3.15

Flow Cytometry

Plots

Confirm that:

☒ The axis labels state the marker and fluorochrome used (e.g. CD4-FITC).

☒ The axis scales are clearly visible. Include numbers along axes only for bottom left plot of group (a 'group' is an analysis of identical markers).

☒ All plots are contour plots with outliers or pseudocolor plots.

☒ A numerical value for number of cells or percentage (with statistics) is provided.

Methodology

Sample preparation

The cells were collected with PBS and the cell suspension was transferred into the tubes, containing 70% ethanol, and fixed for 2 hours. Cells were centrifuged, ethanol was decanted and cells were suspended in 5 ml PBS, and then treated with in 1 ml PI/Triton X-100 staining solution (Propidium iodide (PI)/Triton X-100 staining solution with RNase A, freshly made (To 10 ml of 0.1% (v/v) Triton X-100 (Sigma) in PBS add 2 mg DNase-free RNase A (Sigma) and 200 µl of 1 mg/ml PI) for 30 min at room temperature. Flow cytometry experiments for detecting PI emission were performed in BD FACSCanto II Cell Analyzer (Becton, Dickinson) and the data analysis was performed using DNA content frequency histogram deconvolution software.

Instrument

BD FACSCanto II Cell Analyzer (Becton, Dickinson)

Software

Data were analyzed using FlowJo v7.6

Cell population abundance

All cell lines used in flow cytometry experiments are commercial cell lines. Manufacturers have performed several tests for authenticating cell lines as means of combatting the issues of misidentification and cross contamination of the cultures.

Gating strategy

The gaiting strategy was based, first, on the morphology of viable cells (excluding cell death), comparing relative size (FSC-A) and complexity (SSC-A) of lung cancer cells. Then with FSC-A and FSC-H parameters we detected singlets cells to exclude doublets.

Finally, with PI-A and PI-W parameters we detected the DNA content of stained cells with Propidium Iodide (PI).

☒ Tick this box to confirm that a figure exemplifying the gating strategy is provided in the Supplementary Information.
